# Multi-Omics Insights into the Impact of Fermented Wheat Bran-Soybean Meal-*Broussonetia papyrifera* Mixture Substance on the Gut Microbiota of Late Gestation Sows In Vitro

**DOI:** 10.3390/ani15213199

**Published:** 2025-11-03

**Authors:** Lele Fu, Yushi Chen, Yantao Li, Cheng Wang

**Affiliations:** Xianghu Laboratory, Hangzhou 311231, China; fulele@xhlab.ac.cn (L.F.); chenyushi@xhlab.ac.cn (Y.C.); lyt906040062@163.com (Y.L.)

**Keywords:** fermentation, late gestation, gut microbiota, nutritional value, metabolic functions

## Abstract

**Simple Summary:**

Strategic nutritional interventions during the late gestational period constitute a critical determinant for optimizing maternal-fetal health outcomes. Fermented diets are increasingly recognized as a promising dietary strategy for gestating sows. This in vitro fermentation study demonstrates that bacterial-enzymatic co-fermentation of a wheat bran–soybean meal–*Broussonetia papyrifera* mixed substrate enhances microbial substrate utilization and increases short-chain fatty acid production through the following mechanisms: (1) reduction in dietary fiber content and (2) enrichment of flavor compounds and bioactive metabolites. These findings provide a foundation for developing dietary strategies to improve maternal health during late gestation.

**Abstract:**

Comprehensive maternal nutritional interventions, particularly during late gestation, enhance perinatal outcomes and support long-term maternal-offspring health by modulating the microbiota. Fermented diets are recommended for inclusion in dietary guidelines during gestation, yet the specific metabolites after fermentation and their specific regulatory effects on gut microbiota during late gestation remain unclear. This study investigates the functional benefits of a fermented wheat bran–soybean meal–*Broussonetia papyrifera* mixed substrate (FMS) on the late-gestation gut microbiota using an in vitro fermentation model. The FMS was first fermented for 72 h with bacterial and enzymatic agents (2% *v*/*v*), then anaerobically incubated with fecal inocula from Jinhua pigs. Fermentation significantly enhanced nutritional profiles, increasing crude protein and amino acids while reducing fiber components (neutral detergent fiber, acid detergent fiber, and non-starch polysaccharide, *p* < 0.05). Metabolome analysis revealed a significant increase in the abundance of organic acids, amino acids, and short peptides in FMS, along with the enrichment of D-amino acid and sphingolipid pathways (*p* < 0.05). In addition, FMS significantly increased the abundance of *Limosilactobacillus* and *Lactobacillus*, as well as short-chain fatty acids production, compared to the unfermented group (*p* < 0.05). These findings demonstrate that fermentation pretreatment reduces fiber components, enhances flavor compounds and bioactive metabolites, thereby optimizing microbial utilization and increasing short-chain fatty acids production.

## 1. Introduction

During gestation, pregnant females undergo significant physiological changes driven by elevated energy and nutrient requirements to sustain fetal development and prepare for subsequent lactation [1]. In addition, studies have revealed that late gestation experiences significant catabolic stress, leading to increased oxidative stress and metabolic dysfunction [2,3]. The implementation of maternal comprehensive nutritional and health interventions before conception and throughout pregnancy has been shown to promote optimal fetal growth and enhance obstetrical outcomes [4]. Additionally, maternal diet during pregnancy plays a crucial role in shaping the neonatal microbiome [5]. A child cohort study further demonstrated that breast milk may transfer microbes to the infant gut, thereby influencing the development of their microbiota [6]. Thus, these findings underscore the importance of optimizing nutritional strategies during gestation to support maternal health and fetal development.

Fermentation foods exert beneficial effects on host physiology through bioactive peptides and microbial metabolites, potentially improving cardiovascular, immune, and metabolic functions [7]. Studies have found that fermented feed products have been implemented in the livestock and poultry industry to improve growth performance, reduce overall disease pressure, such as post-weaning diarrhea, and oxidative stress [8,9]. Furthermore, Yu et al. have reported that a *Bacillus licheniformis*-fermented substrate administered during gestation and lactation reduced the milk fat content while improving the piglet body weight [10]. Our previous investigation demonstrated that a fermented substrate comprising corn, soybean meal (SBM), and wine lees administered during late gestation and lactation confers protective effects against intestinal inflammation in offspring by increasing the abundance of *Lactobacillus* [11]. However, the specific metabolites after fermentation and their specific regulatory effects on gut microbiota during late gestation are not well understood.

Soybean meal (SBM) and wheat bran (WB) are important sources of protein and roughage. But their utilization is limited by anti-nutritional factors, high fiber content, and poor digestibility [12,13]. *Broussonetia papyrifera* (BP) is a promising new forage resource with rich bioactive compounds (including alkaloids and flavonoids), offering abundant crude protein, digestible fiber, and minerals [14]. Additionally, it has been reported that supplementation with 300 g/t BP leaf extract can increase growth performance and antioxidant capacity of weaned piglets, reduce the occurrence of diarrhea, enhance immune functions and disease resistance, and affect the composition of fecal microflora [15]. Various methods are used to improve the nutritional value of feed raw material, such as toasting, extrusion, or sieve separation (reducing the level of fiber). One of the more popular methods is fermentation, during which microbes break down anti-nutritional substances, induce the synthesis of various bioactive compounds, primarily antioxidants, reduce the content of raw fiber, and modify the protein amino acid profile [16,17]. Studies have found that dietary supplementation with 4% fermented SBM in the basal maternal diet alleviates oxidative stress in sows during gestation and lactation, and improves the average body weight of their offspring [18]. In addition, He et al. also found that 5% fermented WB could improve the growth performance, immune levels, and intestinal microflora in growing-finishing pigs [19]. Moreover, it has been reported that supplementation with 15% BP silage in the diet could improve the body weight, dry matter intake, and feed conversion rate of ruminant cattle [20]. Thus, we hypothesized that fermentation of the wheat bran–soybean meal–*Broussonetia papyrifera* mixed substrate would enhance its nutritional profile and, consequently, exert more beneficial effects on the gut microbiota of late-gestating sows compared to the unfermented mixture.

This study comprehensively investigates the effects of fermentation substances on late-gestating gut microbiota. Firstly, the change in nutrients and metabolites before and after fermentation of wheat bran–soybean meal–*Broussonetia papyrifera* mixed substrate was analyzed. Subsequently, the regulatory effects of mixture substances with and without fermentation on late gestation gut microbiota were compared via in vitro fermentation. Finally, the correlation analysis was used to reveal the relationship between nutritional characteristics and microbial abundance. This evidence can enhance the use of fermented plant-based diet in late pregnancy from a microbiome and metabolomics perspective.

## 2. Materials and Methods

### 2.1. Fecal Slurry

Six Jinhua sows in late gestation (first-parity, around 14 weeks of gestation), with no prior antibiotic use, provided fecal samples as individual inocula for this study. The sows were housed at Dayanhe Agricultural and Animal Husbandry Farm (Jinhua City, Zhejiang Province, China). All sows were fed a fortified corn–soybean meal-based gestation diet (see Table S1 for ingredients). The diet provided approximately 13.53% crude protein, 3230 kcal/kg digestible energy, and 0.64% lysine. Feed was provided ad libitum (6:00, 12:00, and 18:00), and all sows were maintained under identical housing and management conditions. Fecal samples were collected in the morning (between 08:00 and 10:00). Fresh samples were captured using a clean bag positioned at the anus immediately upon defecation to capture naturally expelled material. When not captured in time, samples were promptly collected from the clean floor within seconds of expulsion, ensuring no contamination from urine. After collection, fresh fecal samples were weighed and mixed with sterile phosphate-buffered saline at 10% (*w*/*v*). Then, the fecal slurry was immediately frozen by immersion in liquid nitrogen, transported to the laboratory under dry ice, and stored at −80 °C until use in fermentation experiments.

### 2.2. Preparation and Nutritional Composition Analysis of Fermented Wheat Bran–Soybean Meal–Broussonetia papyrifera Mixed Substrate (FMS)

**Figure 1:**
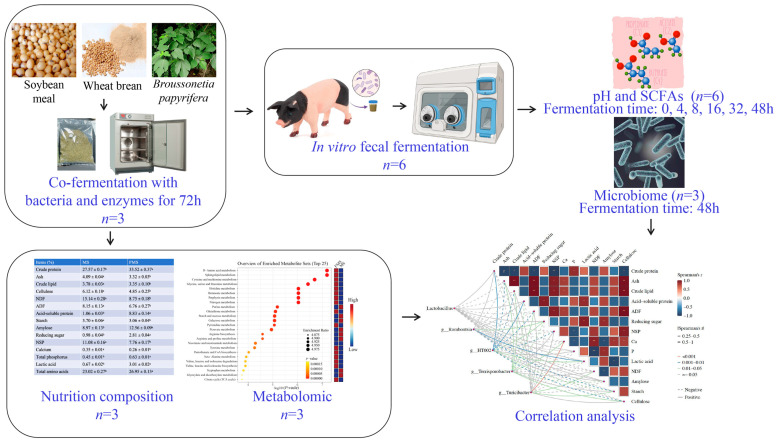
Schematic representation of the experimental design and workflow.

The study design and procedural flow are summarized in Figure 1. The FMS was prepared as previously described [11], with minor modifications. Briefly, sterilized water was added to the ground Wheat Bran–Soybean Meal–*Broussonetia papyrifera* mixture (35%, 35% and 30%) to achieve the optimum moisture content of 40% (*w*/*w*). Commercial microbial and enzyme preparations (including protease, xylanase, *Bacillus subtilis*, *Saccharomyces cerevisiae*, *Pediococcus pentosus* et al., Table S2) are inoculated into the fermentation system at a dose of 0.2%. Following inoculation, the mixture was dispensed into anaerobic fermentation bags and fermented in the incubator (LRH-250, Bluepard, Shanghai, China) at 37 °C for 72 h.

Samples were taken immediately after fermentation for microbial and pH testing, as previously described by Wang et al. [11]. Briefly, 1 g of the sample was mixed with 19 mL of sterile water, and the pH was determined with a pH meter (FE28-Standard, Mettler Toledo, Greifensee, Switzerland). The remaining samples were dried in an electric thermostatic drying oven (50 °C, 8 h, DHG-9140A, Bluepard, Shanghai, China) for nutritional composition analysis and metabolomics analysis.

### 2.3. Simulated In Vitro Digestion of FMS

In vitro oral, stomach, and small intestinal digestion was simulated as described previously [21]. The salivary-simulating fluid, gastric-simulating fluid, and small-intestine-simulating fluid consisted of the corresponding electrolytes, enzymes, and water. Briefly, 25 g of fermented feed was suspended in 300 mL PBS, and 2.25 mL of α-amylase was added for 15 min at 37 °C. Then, the pH was adjusted to 2.5 ± 0.1 with 2 M HCl, and 1 mL pepsin (10%) was added, followed by 30 min incubation at 37 °C. Finally, 50 mL sodium maleate buffer (0.1 M) was added, pH adjusted to 6.9 ± 0.1 with 2 M NaOH, and enzymes (50 mL trypsin, 12.5%; 2 mL amyloglucosidase) were introduced before a 3 h incubation at 37 °C. After freeze-drying, the samples were stored at −80 °C before the in vitro fermentation experiments.

### 2.4. In Vitro Fermentation

Fermented and unfermented substrates were subjected to in vitro fermentation (designated as FMS-F and MS-F groups). For each in vitro fermentation, 4 g of substrates (freeze-dried powders) were suspended in a 100 mL sterile nutrient basal medium (Table S3). Then, 2.5 mL of suspension was inoculated into each fermentation tube with 2.5 mL of fecal slurry. The fermentation tubes were incubated and placed in an anaerobic hood (10% H_2_, 10% CO_2_, 80% N_2_, 37 °C, AW 500TG, Electrotek, West Yorkshire, UK) with shaking (140 rpm). The control group (CON) only contained basal medium and fecal suspension. Sampling was performed at 0 h, 4 h, 8 h, 16 h, 32 h, and 48 h, after which the pH (FE28-Standard, Mettler Toledo, Greifensee, Switzerland) was immediately tested. Additionally, the fermented materials for 16S rRNA analysis and SCFA analysis were stored (−80 °C) until analysis.

### 2.5. 16S rRNA Analysis

The 48 h in vitro fermentation time point was selected for 16S rRNA gene sequencing based on the kinetic profiles of SCFA production, which indicated that microbial fermentation had reached a relatively stable phase by 48 h. The Shanghai Majorbio Bio-Pharm Technology Co., Ltd. (Shanghai, China) performed the 16S rRNA gene sequencing procedure. The hypervariable region V3–4 of the bacterial 16S rRNA gene was amplified with primer pairs 338F (5′-ACTCCTACGGGAGGCAGCAG-3′) and 806R (5′-GGACTACHVGGGTWTCTAAT-3′) [22]. The raw sequencing data were processed and analyzed according to established protocols, as described by Wang et al. [23]. Sequences were submitted to the NCBI Sequence Read Archive (SRA) database (BioProject: PRJNA1234503).

### 2.6. Metabolomic Profiling by LC-TOF/MS

The metabolomic analysis was performed by Shaanxi Techshake Biotechnology Co., Ltd. (Xi’an, China) [24], which included sample preparation, LC-TOF/MS data acquisition, and initial processing. The obtained data were applied to sparse partial least squares discriminant analysis (sPLS-DA) by the R package *ropls* (v1.22.0) [25]. Differential metabolites were identified with variable importance projection (VIP) > 1.0 and *p* < 0.05. To further interpret the biological relevance of the metabolites, metabolic pathway analyses and KEGG enrichment analysis were performed by an online analysis platform in MetaboAnalyst version 6.0 (https://www.metaboanalyst.ca, accessed on 15 January 2025) [26].

### 2.7. Quantification of Short-Chain Fatty Acid (SCFA) Profiling

Briefly, 1 mL of the fermentation slurry was centrifuged (4 °C, 20,000× *g*, 15 min), and 200 μL of supernatant was then diluted with 25% metaphosphoric acid (5:1, *v*/*v*) and incubated at 4 °C for 30 min. Finally, the supernatant was obtained and filtered after centrifugation (4 °C, 12,000× *g*, 15 min) and used for SCFA analysis by gas chromatography (GCMS-TQ8050 NX, Shimadzu, Kyoto, Japan).

### 2.8. Statistical Analysis

All data are expressed as mean ± SEM (Standard Error of the Mean). Statistical analyses were performed using a two-tailed Student’s *t*-test, with statistical significance defined as *p* < 0.05. Graphical representations were generated using GraphPad Prism 10.0 (GraphPad, San Diego, CA, USA). Significant differences in microbial genera and characteristic taxa were determined through the Wilcoxon rank-sum test and linear discriminant analysis effect size (LEfSe). The Mantel test was employed to assess correlations between metabolites, nutritional composition, and microbiota. Interrelationships among differentially abundant bacterial taxa were evaluated using Spearman’s correlation analysis.

## 3. Results

### 3.1. Nutrition Composition Changes After Fermentation

As we suspected, the pH of the feed was significantly reduced after fermentation (from 6.74 ± 0.01 to 4.9 ± 0.03). The content of crude protein, acid-soluble protein, amylose, reducing sugar, total phosphorus, and lactic acid is significantly increased after fermentation (*p* < 0.05), while cellulose, neutral detergent fiber (NDF), acid detergent fiber (ADF), non-starch polysaccharide (NSP), and starch are remarkably decreased (Table 1, *p* < 0.05). In addition, fermentation significantly increased the contents of total amino acids, including threonine, valine, methionine, leucine, lysine, serine, tryptophan, alanine, tyrosine, glycine, proline, and glutamic acid (Table 2, *p* < 0.05).

### 3.2. Metabolic Changes After Fermentation

**Figure 2:**
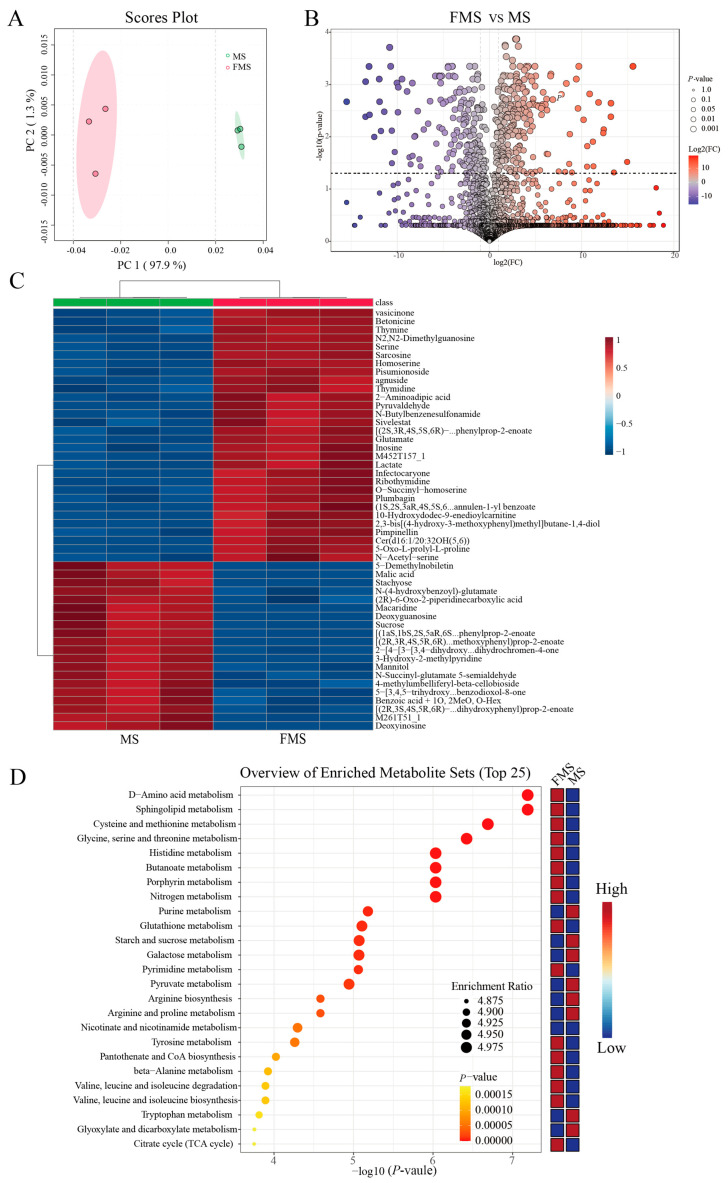
Metabolic changes after fermentation *(n* = 3). (**A**) PCA plot of metabolites between fermented mixture substrate group (FMS) and unfermented mixture substrate group (MS). (**B**) Volcano plot of FMS vs. MS. Differentially abundant metabolites were defined as those with variable importance in the projection (VIP) > 1.0 from orthogonal partial least squares-discriminant analysis (OPLS–DA) and *p* < 0.05. (**C**) Hierarchical clustering heatmap visualizing the top 25 differentially expressed metabolites between FMS and MS (VIP > 1.0 and *p* < 0.05). Rows represent metabolites, columns represent samples, and color intensity reflects relative abundance. (**D**) Metabolic pathway enrichment analysis (TOP 25 most enriched pathways) based on differentially abundant metabolites.

The principal component analysis (PCA) plot visually demonstrated the within-group similarity and between-group differences (Figure 2A). In addition, the cluster analysis shows that the samples before and after fermentation are grouped into 2 clusters (Figure S1). These results suggested that there were significant changes in metabolism after fermentation.

The volcano plot visually showed the overall distribution of differentiated metabolites between groups. There were 219 metabolites significantly up-regulated and 164 significantly down-regulated after fermentation (*p* < 0.05, cutoff for logFC is 1, Figure 2B). The top 50 significantly altered metabolites during fermentation are shown in Figure 2C. The abundance of organic acids, amino acids, and short peptides such as glutamic acid, threonine, serine, sarcosine, and L-homoserine was higher in the fermentation group, while the sucrose, stachyose, and mannitol were significantly higher in the unfermented group.

Then, the enrichment analysis of differential metabolites identified the top 25 metabolic pathways involved during fermentation (Figure 2D and Table S4). Before fermentation, purine metabolism, glyoxylate and dicarboxylic acid metabolism, starch and sucrose metabolism, and galactose metabolism were remarkably enriched. After fermentation, D-amino acid metabolism, sphingolipid metabolism, glycine, serine and threonine metabolism, cysteine and methionine metabolism, histidine metabolism, butyric acid metabolism, arginine and proline metabolism, valine, leucine and isoleucine degradation and biosynthesis, and pyrimidine metabolism were significantly enriched.

### 3.3. Changes in pH Value and SCFAs Content During In Vitro Fermentation

It has been found that fermentation could change the nutritional compositions and metabolites. However, it was still not clear whether the FMS was more conducive to the utilization of the microbial community of sows in late pregnancy. Thus, we investigated the in vitro digestion and fermentation properties of fermented Wheat Bran–Soybean Meal–*Broussonetia papyrifera* mixture.

**Figure 3:**
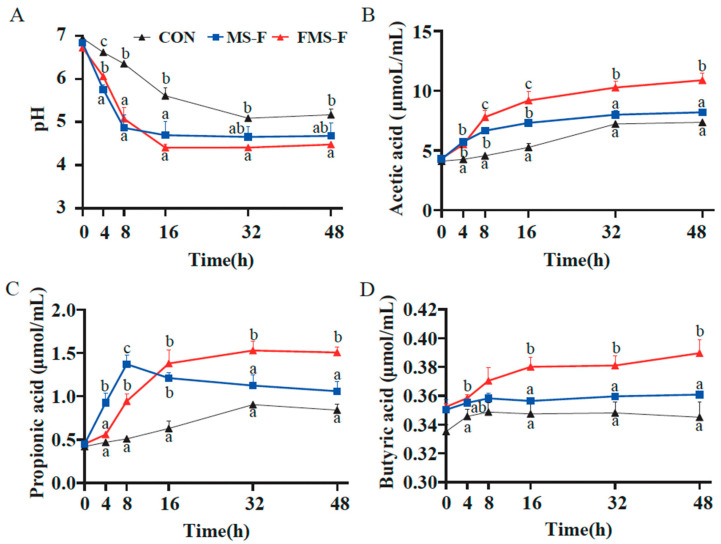
Changes in pH value and short-chain fatty acid (SCFA) content during in vitro fermentation (*n* = 6). (**A**) Dynamics of pH over time during in vitro fermentation for the control (CON) group, the fermented mixed substrate (FMS-F) group, and the unfermented mixed substrate group (MS-F). (**B**) Acetate, (**C**) propionate, and (**D**) butyrate concentrations at 0, 4, 8, 16, 32, and 48 h post-incubation. Data are represented as means ± SEM. Different lowercase letters indicate significant differences among groups within the same time point (*p* < 0.05). Lines represent group means with distinct styles and colours indicating different treatment groups, as defined in the legend of panel (**A**).

Acid production was reflected in decreased pH during the fermentation process (Figure 3A). Compared with the CON (6.94 to 5.17), the MS-F group and FMS-F group decreased significantly from the initial pH 6.78 to the lowest point 4.68 and 4.48, respectively, (*p* < 0.05). The change trend of pH in the FMS-F group was consistent with that in the MS-F group in the initial 8 h. Interestingly, pH in the FMS-F group still had a significant decline from 8 h to 16 h, while the MS-F group had begun to stabilize.

The decrease in pH may be related to the production of SCFAs. Here, it was found that the FMS-F group had a significantly higher level of acetic acid, propionic acid, and butyric acid than the MS-F group after anaerobic fermentation for 48 h (*p* < 0.05, Figure 3B–D). The SCFAs accumulated quickly in the initial 16 h during the fermentation. Interestingly, the MS-F group had a significantly higher level of propionic acid in the first 8 h of fermentation compared to the FMS-F group, and then the concentration decreased. The above results indicated that FMS-F had a prebiotic effect by significantly promoting acid production.

### 3.4. Microbial Community Composition After In Vitro Fermentation

**Figure 4:**
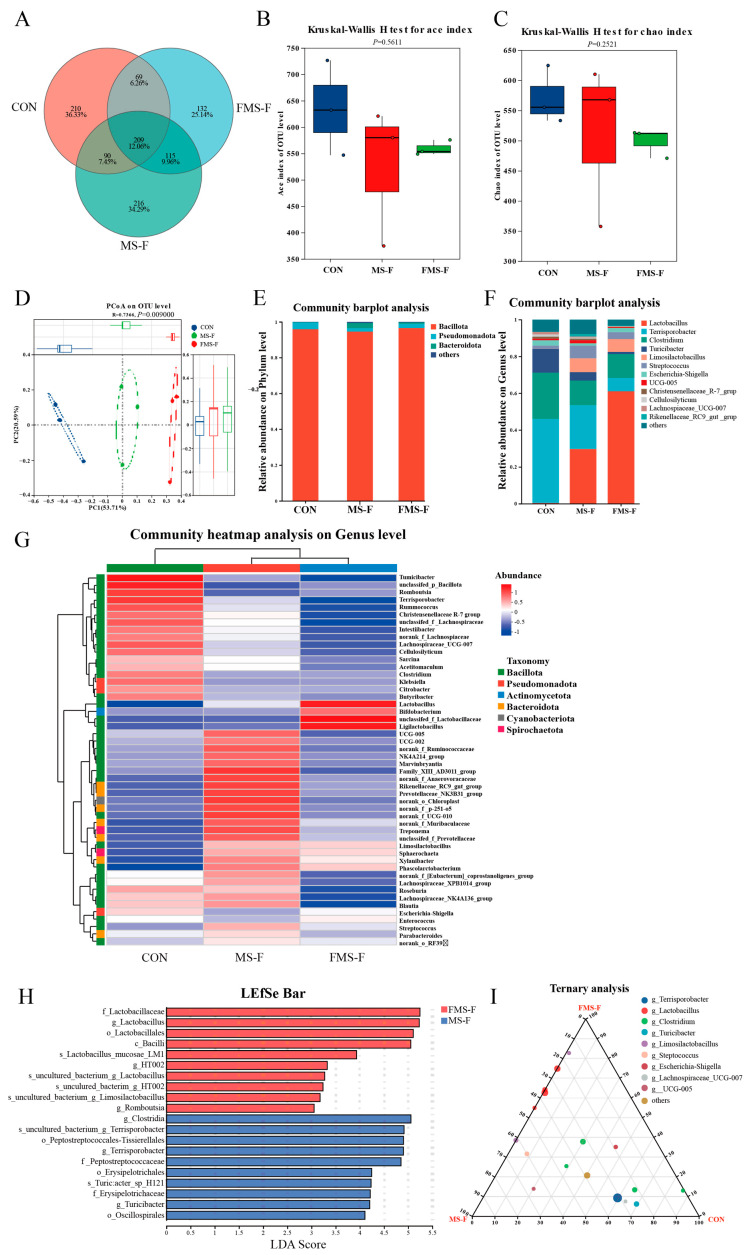
Microbial community composition and variations after 48 h in vitro fermentation (*n* = 3). (**A**) Venn diagram showing shared and unique operational taxonomic units (OTUs) among the control (CON), fermented mixed substrate (FMS-F), and unfermented mixed substrate (MS-F) groups after in vitro fermentation. (**B**) Ace index of α-diversity. (**C**) Chao index of α-diversity. (**D**) Principal coordinates analysis (PCoA) based on weighted UniFrac distances (*p* < 0.01). (**E**) Bar plots at the phylum level. (**F**) Bar plots at the genus level. (**G**) Community heatmap analysis on the genus level. (**H**) Linear discriminant analysis (LDA) effect size (LEfSe) identifying taxonomic biomarkers differentially enriched between FMS-F and MS-F (LDA score > 3.0, *p* < 0.05). (**I**) Ternary plot visualizing the relative abundance distribution of dominant genera among CON, FMS-F, and MS-F groups.

A Venn diagram showed the OTU overlap observed in these samples. There are 209 shared OTUs in three groups, with 210 specific OTUs in the CON, and the specific OTU numbers of the MS-F and FMS-F groups are 216 and 132, respectively, (Figure 4A). The richness and evenness of microbial community structure can be reflected by alpha-diversity. However, there was no significant difference in the Ace index and the Chao index among groups (Figure 4B,C). Principal coordinates analysis (PCoA) shows that the β-diversity of the microbiota is significantly different among the groups (Figure 4D, *p* = 0.009). Thus, the regulation of FMS-F could change the structure of gut microbiota.

To investigate the effects of in vitro fermentation on gut microbiota structure, the 16S rRNA is performed after 48 h of fecal incubation. The results show that the Bacillota is the dominant microbiota of the bacterial community at the phylum level (Figure 4E). While at the genus level, *Lactobacillus*, *Terrisporobacter,* and *Clostridium* are the largest proportion in the entire microbial community (Figure 4F). Notably, the *Lactobacillus* and *Limosilactobacillus* were hardly present in the CON. In addition, the heatmap shows the top 50 microbes at the genus level, and the abundance of Lactobacillus, Bifidobacterium, unclassifed_f_*Lactobacillaceae,* and *Ligilactobacillus* were higher in the FMS-F group (Figure 4G).

The histogram of linear discriminant analysis (LDA) value distribution is shown in Figure 4H, revealing the biomarkers with statistical differences between MS-F and FMS-F. The FMS-F group had a high abundance in the genera of *Lactobacillus* and *Romboutsia*, while the MS-F group had a higher abundance in *Turicibacter* and *Terrisporobacter*. In addition, the ternary plot showed that the *Lactobacillus* and *Limosilactobacillus* existed mainly in MS-F and FMS-F groups (Figure 4I).

### 3.5. Correlation Analysis

**Figure 5:**
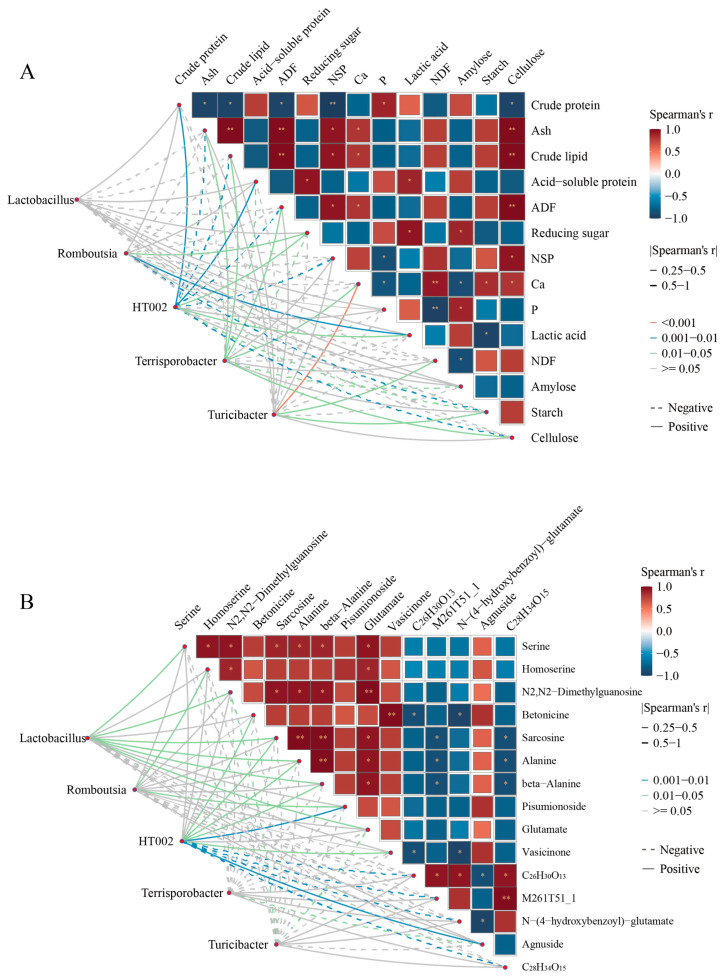
Correlation analysis between metabolites, nutritional composition, and microbiota. (**A**) Correlation analysis between nutritional composition and microbiota. (**B**) Correlation analysis between metabolites and microbiota. Pairwise nutrition composition or metabolites comparisons are shown, with a color gradient denoting Spearman’s correlation coefficients. Line colors (pink: *p* <0.001, blue: *p =* 0.001–0.01, green: *p* = 0.05–0.05) indicate varying strengths of correlation; gray lines show no correlation (*p* > 0.05). Line thickness reflects the magnitude of Spearman’s correlation coefficient, with thicker lines indicating stronger associations. Solid and dotted lines represent positive and negative correlations. * *p* < 0.05, ** *p* < 0.01.

Mantel’s test reveals significant correlations between distinguished microbiota and nutritional composition or metabolites (Figure 5A, B, and Tables S5–S8). The results showed that *Romboutsia* exhibited a positive correlation with reducing sugar, lactic acid, homoserine, and pisumionoside, whereas it was negatively correlated with starch. HT002 was positively correlated with crude protein, acid–soluble protein, betonicine, sarcosine, alanine, beta-alanine, pisumionoside, vasicinone, and agnuside, but negatively associated with NSP, cellulose, and N−(4−hydroxybenzoyl)−glutamate. *Terrisporobacter* displayed positive correlations with ash, crude lipid, ADF, Ca, and cellulose, while showing negative correlations with reducing sugar, amylose, and agnuside. *Turicibacter* was positively linked to NDF and starch, but negatively linked to N_2_, N_2_-dimethylguanosine, and glutamate. *Lactobacillus*, on the other hand, was positively correlated with serine, homoserine, N_2_, N_2_-dimethylguanosine, sarcosine, alanine, beta-alanine, pisumionoside, and glutamate.

## 4. Discussion

The fermentation significantly altered the nutritional composition and metabolites of the feed. The microbiota rapidly produced beneficial metabolites and active substances such as lactic acid during fermentation, thus reducing the pH value of its surrounding environment [27,28]. It has been reported that the crude protein and acid-soluble protein significantly increased after the fermentation process [29,30]. This increase may result from two mechanisms. First, microbial respiration consumes organic matter, potentially leading to a relative increase in protein concentration due to partial dehydration or volume reduction. Second, microorganisms can assimilate inorganic nitrogen and carbon sources to synthesize bacterial biomass and produce microbial proteins [31], further contributing to higher total protein levels. However, this outcome is not universal and depends on microbial composition and substrate properties. For instance, natural fermentation of cassava leaves resulted in a 58% decrease in protein content after 36 h, attributed to proteolytic degradation into peptides and free amino acids [32]. Similarly, inoculation with specific strains leads to divergent effects; *Bacillus amyloliquefaciens*-inoculated whole-plant corn silage showed reduced crude protein content, whereas *Bacillus subtilis*-inoculated silage exhibited increased crude protein levels [33]. These discrepancies highlight the variability in outcomes due to differences in fermenting substrates, inoculations, and product characteristics across studies.

Additionally, studies indicate that some soy proteins in SBM exist in an inactive form that needs to be released through fermentation [34]. The increased acid-soluble protein content reflects the production of bioactive peptides during fermentation and is closely associated with protein desensitization, physiological activity, and improved digestibility [30]. Our results suggested that the fermentation effectively enhances the nutritional and functional properties of protein sources.

Studies have shown that *Bacillus subtilis* and *Saccharomyces cerevisiae* can produce large amounts of cellulase [35,36], which was consistent with our results that the contents of NDF and ADF were significantly decreased after fermentation. Wang et al. reported that compared with the addition of cellulase alone, the NDF and ADF content of mixed silage of whole-plant corn and peanut vines with *Lactobacillus plantarum* and cellulase was reduced more [37]. Thus, there was a synergistic effect of different inoculations on fermentation.

Co-fermentation with bacteria and enzymes facilitates proteolysis, yielding low-molecular-weight peptides and amino acids that serve as precursors for flavor compound synthesis [27]. Multiple studies have reported an increase in total and essential amino acid content after fermentation of legumes and cereals [38,39]. Solid-state fermentation of soybean with a *Bacillus subtilis* and *Aspergillus oryzae* coculture improved the level of most essential amino acids, whereas *Rhizopus oryzae* fermentation only resulted in significantly higher levels of threonine [40]. Other studies have reported either no significant changes or a reduction in the essential amino acid content during the fermentation of pea protein when utilizing a single strain [24,41]. Thus, co-fermentation may be more advantageous.

Consistent with previous results, metabolome results showed that the abundances of amino acids and lactate increased significantly after fermentation, while the abundances of sucrose and stachyose decreased significantly. Most microorganisms can use sucrose as a carbon source for growth, and *Bacillus subtilis* can even ferment sucrose to produce poly-gamma-glutamic acid [42,43]. Stachyose is a prebiotic to enhance the health-conferring microbes such as *Bifidobacteria* and *Lactobacilli* [44].

KEGG pathway analysis revealed the main metabolic routes affected by the significantly altered metabolites, providing insights into the underlying mechanisms of fermentation. The present study showed that D-amino acids metabolism, Sphingolipid metabolism, Glycine, serine and threonine metabolism, Histidine metabolism, and Cysteine and methionine metabolism were the top five enriched metabolism pathways during fermentation. Studies have confirmed that lactic acid bacteria synthesize multiple D-amino acids during fermentation, contributing to a sweeter flavor profile relative to their L-isomers [45]. These facts suggest that the enhanced D-amino acid metabolism may relate to the improved flavor after fermentation. Most Bacteroidetes and certain alpha-Proteobacteria species synthesize sphingolipids, but fungi such as *Saccharomyces* can also synthesize sphingolipids [46]. Studies highlight the key role of bacterial sphingolipids in sustaining gut homeostasis and reducing systemic inflammatory responses [47]. In addition, amino acid metabolism was enhanced as predicted. All these results revealed an improvement in flavor and bioactive compounds after fermentation.

In vitro fermentation has been developed from human and ruminant studies [48]. Although in vitro fermentation cannot replicate the complex in vivo environment, the conditions are more controllable, making it an effective and widely used means to simulate the animal intestinal environment to study the relationship between substrate and gut microbiota [49,50,51]. The change trend of pH in the MS-F group was consistent with that in the FMS-F group, which suggested that the gut microbiota development grew faster in the early stage, rapidly produced acid, thus reducing the pH value of its surrounding environment. Additionally, the pH values of the final fermented substrate were between 4.0 and 5.0; these results were similar to the studies of Wang et al. [52].

The SCFAs exhibited various physiological activities and played an important role in maintaining intestinal homeostasis. Acetic acid, propionic acid, and butyric acid can be used as an energy source for intestinal epithelial cells as well as regulate mucosal and systemic immunity [53]. Therefore, the analysis of SCFAs is essential for a more comprehensive evaluation of the in vitro fermentation characteristics. Notably, the FMS-F group demonstrated significantly higher SCFA concentrations compared to the MS-F group, indicating superior health benefits. Several microbes, including *Peptostreptococcaceae*, *Turicibacter*, *Romboutsia*, *Clostridium butyricum,* and *Lactobacillus*, have been identified as SCFA-producing bacteria [54,55,56]. *Lactobacillus* is a well-known probiotic that has multiple functions, including modulating metabolism and ameliorating colitis development [57]. Additionally, the *Romboutsia* could regulate the expression of peripheral cytokines such as IL-1 and IFN-γ [58]. Conversely, *Turicibacter* and *Peptostreptococcaceae* were reported to be associated with pro-inflammation in inflammatory bowel disease and colorectal cancer [59,60,61]. Our previous study has also found that the fermented SBM dramatically promotes *Bifidobacterium* and *Lactobacillus* abundance, and enhances the fructose and mannose metabolism function of microorganisms [62]. In addition, the signaling molecules released by certain bacteria are sensed by intracellular receptors or membrane-bound receptors of other members in the community, leading to the collective isogenic signaling molecule synthesis and synchronized activities [63]. The current findings reveal that FMS-F significantly decreased in the relative abundance of gut pathogens (*Turicibacter* and *Peptostreptococcaceae*) while increasing the abundance of probiotics (*Lactobacillus* and *Romboutsia*), suggesting that fermented feed promotes the host’s health through microbiota modulation. Although the sample size for microbiome analysis was small (*n* = 3 per group), it is consistent with established practices in in vitro fermentation studies [49,51], where highly controlled conditions minimize biological variability.

Diet is increasingly recognized as a central factor shaping the composition and functional profile of the gut microbiota among various host-related and environmental influences [64]. Correlation analysis revealed significant relationships between sow gut microbiota and feed metabolites or nutritional composition. Compared with other genera, the *Terrisporobacter* and HT002 showed more correlation with the nutritional composition. The *Lactobacillus* and HT002 showed more correlation with feed metabolites. *Terrisporobacter* plays a crucial role in enabling the intestinal microbiota to promptly adapt to plant-derived dietary substrates through its fiber-degrading activity [65]. Our results confirmed that the degradation of cellulose after fermentation corresponded to a decrease in the abundance of *Terrisporobacter*. Emerging evidence indicates a strong correlation between *Turicibacter* abundance and dietary lipid [66]. However, there was no significant correlation between crude lipid and *Turicibacter* in our study. Thus, the relationship between nutritional composition and the microbiome was complex and influenced by many factors. The positive correlation of probiotics, including *Lactobacillus*, *Romboutsia*, and HT002, with flavor substances (serine, alanine, glutamate, and sarcosine) and bioactive metabolites (betonicine, vasicinone, and agnuside) indicated that amino acids can serve as precursors for the synthesis of SCFA by bacteria [67].

## 5. Conclusions

In conclusion, this study demonstrated that fermentation pretreatment decreased fiber components, enriched flavor compounds, and bioactive metabolites, thereby optimizing microbial utilization and increasing short-chain fatty acids production. The results support co-fermentation with bacteria and enzymes for processing substances as an effective strategy to meet the nutritional requirements of late gestation and enhance gut health. Future enhancements may broaden its use in the diet of sows and further clarify its regulatory effect on maternal and offspring health.

## Figures and Tables

**Table 1:** Nutrition composition of the wheat bran–soybean meal–*Broussonetia papyrifera* mixture substance before and after fermentation.

Items (%)	MS ^1^	FMS ^2^
Crude protein	27.57 ± 0.17 ^b^	33.52 ± 0.37 ^a^
Ash	4.09 ± 0.04 ^a^	3.32 ± 0.03 ^b^
Crude lipid	3.78 ± 0.03 ^a^	3.35 ± 0.10 ^b^
Cellulose	6.12 ± 0.18 ^a^	4.85 ± 0.25 ^b^
NDF	15.14 ± 0.28 ^a^	8.75 ± 0.18 ^b^
ADF	8.15 ± 0.13 ^a^	6.76 ± 0.27 ^b^
Acid-soluble protein	1.06 ± 0.03 ^b^	8.83 ± 0.14 ^a^
Starch	3.70 ± 0.06 ^a^	3.06 ± 0.04 ^b^
Amylose	8.97 ± 0.13 ^b^	12.56 ± 0.09 ^a^
Reducing sugar	0.98 ± 0.04 ^b^	2.81 ± 0.04 ^a^
NSP	11.08 ± 0.16 ^a^	7.76 ± 0.17 ^b^
Calcium	0.35 ± 0.01 ^a^	0.26 ± 0.01 ^b^
Total phosphorus	0.45 ± 0.01 ^b^	0.63 ± 0.01 ^a^
Lactic acid	0.67 ± 0.02 ^b^	3.01 ± 0.02 ^a^

^1^ MS: Wheat bran–soybean meal–*Broussonetia papyrifera* mixed substrate. ^2^ FMS: Fermented wheat bran–soybean meal–*Broussonetia papyrifera* mixed substrate. Data in the table are represented as mean ± SEM, and different letters in the same row indicate significant differences (*p* < 0.05).

**Table 2:** Amino acid composition of the mixture before and after fermentation.

Items (%)	MS ^1^	FMS ^2^
Essential amino acid		
Threonine	0.89 ± 0.01 ^b^	1.15 ± 0.08 ^a^
Valine	1.23 ± 0.01 ^b^	1.31 ± 0.04 ^a^
Methionine	0.27 ± 0.00 ^b^	0.32 ± 0.00 ^a^
Isoleucine	0.91 ± 0.01 ^a^	0.75 ± 0.01 ^b^
Leucine	1.58 ± 0.02 ^b^	2.37 ± 0.05 ^a^
Phenylalanine	1.09 ± 0.05	1.16 ± 0.06
Lysine	1.05 ± 0.02 ^b^	1.50 ± 0.04 ^a^
Histidine	0.52 ± 0.00	0.52 ± 0.01
Arginine	1.08 ± 0.03	1.13 ± 0.09
Tryptophan	0.54 ± 0.02 ^b^	0.72 ± 0.01 ^a^
Non-essential amino acid		
Serine	1.79 ± 0.03 ^b^	2.06 ± 0.08 ^a^
Glutamic acid	5.13 ± 0.13 ^b^	6.37 ± 0.09 ^a^
Glycine	1.66 ± 0.06 ^b^	1.81 ± 0.01 ^a^
Alanine	1.43 ± 0.03 ^b^	1.56 ± 0.04 ^a^
Cysteine	0.31 ± 0.01 ^b^	0.40 ± 0.01 ^a^
Aspartic acid	1.93 ± 0.06	2.05 ± 0.05
Proline	0.79 ± 0.00 ^b^	0.87 ± 0.02 ^a^
Tyrosine	0.82 ± 0.00 ^b^	0.91 ± 0.01 ^a^
Total amino acids	23.02 ± 0.27 ^b^	26.95 ± 0.13 ^a^

^1^ MS: Wheat bran–soybean meal–*Broussonetia papyrifera* mixed substrate. ^2^ FMS: Fermented wheat bran–soybean meal–*Broussonetia papyrifera* mixed substrate. Data in the table are represented as mean ± SEM, and different letters in the same row indicate significant differences (*p* < 0.05).

## Data Availability

The original contributions presented in this study are included in the article. Further inquiries can be directed to the corresponding authors. Sequence data in this study were uploaded to the NCBI SRA database (PRJNA1234503).

## Supplementary Materials

The following supporting information can be downloaded at: https://www.mdpi.com/article/10.3390/ani15213199/s1, Figure S1: Heatmap cluster analysis of different metabolites before and after fermentation (n = 3); Table S1: Ingredient and nutrient composition of gestation diet; Table S2: Composition of microbial and enzyme preparations for fermentation; Table S3: The medium composition for in vitro fermentation; Table S4: Enrichment analysis of differential metabolites reveals key metabolic pathways during fermentation; Table S5: Spearman correlation coefficients (r) and *p*-values among nutrient components of the fermented substrate; Table S6: Spearman correlation coefficients (r) and *p*-values (*p*) between nutrient components and microbial taxa or metabolites; Table S7: Spearman correlation coefficients (r) and *p*-values among metabolisms of the fermented substrate; Table S8: Spearman correlation coefficients (r) and *p*-values (*p*) between microbiota and metabolites.
