# Supplementary material for: Multi-Omics Insights into the Impact of Fermented Wheat Bran-Soybean Meal-Broussonetia papyrifera Mixture Substance on the Gut Microbiota of Late Gestation Sows In Vitro
Source: Animals (Basel). 2025 Nov 3;15(21):3199. doi: 10.3390/ani15213199 (PMC12609904; doi:10.3390/ani15213199)
Supplement: Supplementary file 1 [file animals-15-03199-s001.zip › animals-3873050-supplementary.pdf]

Supplementary

Figure S1 Heatmap cluster analysis of different metabolites before and after fermentation.

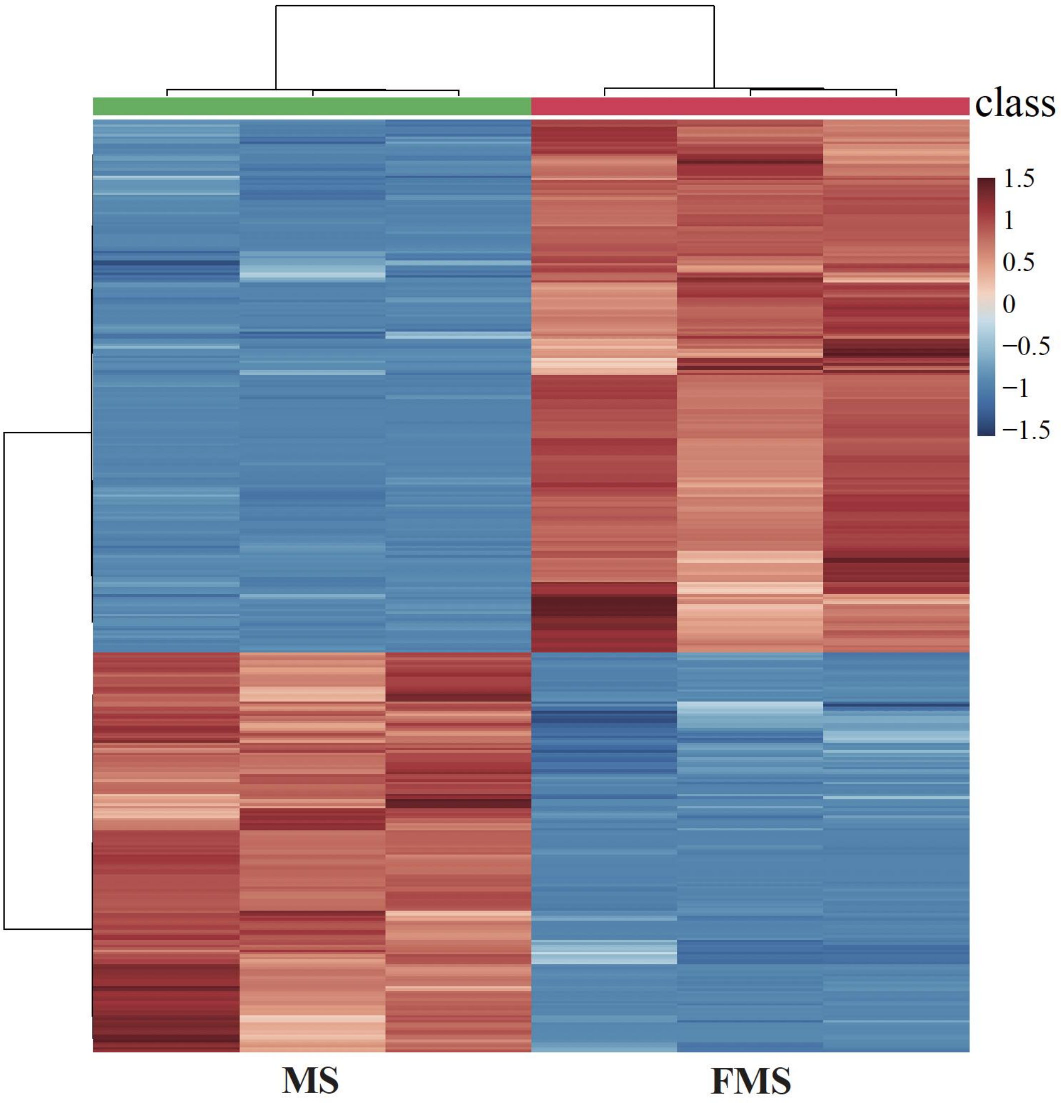

Table S1 Ingredient and nutrient composition of gestation diet

Ingredient, %	
Corn	66.00
Soybean meal	15.00
Wheat bran	14.23
Soybean oil	1.80
Limestone	0.81

Dicalcium phosphate	1.35
Sodium chloride	0.40
Choline chloride	0.15
Vitamin premix[1]	0.04
Mineral premix[2]	0.20
Enzyme preparation	0.02
Nutrient composition	
Digestible energy, kcal/kg	3230
Crude protein	13.54
Crude fiber	3.37
Calcium	0.74
Available phosphorus	0.35
Total lysine	0.64
Total methionine	0.23

[1]Vitamin premix provided per kilogram of diet: Vitamin A 9600 IU; Vitamin D3 1920 IU; Vitamin E 80 IU; Vitamin K3 3.84 mg; Vitamin B1 1.6 mg; Vitamin B2 5.76 mg; Vitamin B6 2.88 mg; Vitamin B12 0.02 mg; Pantothenic acid 20 mg; Biotin 0.384 mg; Folic acid 3.2 mg; Niacin 32 mg.

[2]Mineral premix provided per kilogram of diet: Cu 12 mg; Fe 100 mg; I 0.2 mg; Zn 120 mg; Mn 28 mg; Se 0.2 mg.

Table S2 Composition of microbial and enzyme preparations for fermentation

Items	per gram
Protease	≥ 50000 U[1]
Xylanase	≥ 50000 U
Bacillus subtilis	≥ 5×10^8 CFU[2]
Saccharomyces cerevisiae	≥ 7×10^8 CFU
Lactobacillus plantarum	≥ 2×10^8 CFU
Pediococcus pentosus	≥ 6×10^8 CFU

U[1]: Active unit; CFU[2]: Colony-Forming Units

Table S3 The medium composition for *in vitro* fermentation

Items	per Litre

Peptone	0.2g
Yeast extract	0.2g
$NaHCO_3$	0.2g
Bile acids	0.25g
L-cysteine hydrochloride	0.25g
K_2HPO_4	0.4g
$CaCl_2 \cdot 2H_2O$	0.25g
$MgSO_4 \cdot 7H_2O$	0.5g
Hemin	0.01g
NaCl	2.5g
Vitamin K	10μL
Tween 80	2mL
Wolfe's mineral solution	10mL
ddH_2O	to 1L
pH	7.0

Table S4 Enrichment analysis of differential metabolites reveals key metabolic pathways during fermentation

	Total Cmpd	Hits	Statistic Q	Expected Q	Raw p	Holm p	FDR
D-Amino acid metabolism	15	1	99.959	20	6.46E-08	1.94E-06	9.68E-07
Sphingolipid metabolism	32	1	99.959	20	6.46E-08	1.94E-06	9.68E-07
Cysteine and methionine metabolism	33	2	99.958	20	2.05E-07	5.73E-06	2.05E-06
Glycine, serine and threonine metabolism	33	3	99.851	20	3.78E-07	1.02E-05	2.84E-06
Histidine metabolism	16	1	99.843	20	9.27E-07	2.41E-05	3.48E-06
Butanoate metabolism	15	1	99.843	20	9.27E-07	2.41E-05	3.48E-06
Porphyrin metabolism	31	1	99.843	20	9.27E-07	2.41E-05	3.48E-06
Nitrogen metabolism	6	1	99.843	20	9.27E-07	2.41E-05	3.48E-06
Purine metabolism	70	6	99.136	20	6.60E-06	0.00014514	2.01E-05
Glutathione metabolism	28	3	99.452	20	7.85E-06	0.00016482	2.01E-05
Starch and sucrose metabolism	18	1	99.524	20	8.51E-06	0.00017012	2.01E-05
Galactose metabolism	27	3	99.523	20	8.55E-06	0.00017012	2.01E-05
Pyrimidine metabolism	39	3	98.662	20	8.70E-06	0.00017012	2.01E-05
Pyruvate metabolism	23	2	99.458	20	1.14E-05	0.00019373	2.44E-05
Arginine biosynthesis	14	3	98.284	20	2.61E-05	0.00041733	4.89E-05
Arginine and proline metabolism	36	4	98.284	20	2.61E-05	0.00041733	4.89E-05
Nicotinate and nicotinamide metabolism	15	1	98.841	20	5.06E-05	0.00070812	8.93E-05
Tyrosine metabolism	42	2	98.76	20	5.49E-05	0.00071344	9.15E-05
Pantothenate and CoA biosynthesis	20	2	98.22	20	9.47E-05	0.0011361	0.00014948
beta-Alanine metabolism	21	1	98.224	20	0.00011906	0.0013096	0.00017484

Valine, leucine and isoleucine degradation	39	1	98.157	20	0.00012821	0.0013096	0.00017484
Valine, leucine and isoleucine biosynthesis	8	1	98.157	20	0.00012821	0.0013096	0.00017484
Tryptophan metabolism	41	1	97.98	20	0.00015412	0.0013096	0.00020102
Glyoxylate and dicarboxylate metabolism	31	4	97.477	20	0.00017654	0.0013096	0.00021331
Citrate cycle (TCA cycle)	20	2	97.473	20	0.00017776	0.0013096	0.00021331
Glycerophospholipid metabolism	36	1	97.389	20	0.00025799	0.0013096	0.00028666
Ether lipid metabolism	20	1	97.389	20	0.00025799	0.0013096	0.00028666
Alanine, aspartate and glutamate metabolism	28	4	96.813	20	0.00038325	0.0013096	0.00041062
Pentose phosphate pathway	23	1	96.514	20	0.00046121	0.0013096	0.00047712
Caffeine metabolism	10	1	95.877	20	0.00064642	0.0013096	0.00064642

Table S5 Spearman correlation coefficients (r) and P-values among nutrient components of the fermented substrate

Row names	Column names	r	P
Crude protein	Crude protein	1.00	0.00
Ash	Crude protein	-0.94	0.02
Crude lipid	Crude protein	-0.94	0.02
Acid-soluble protein	Crude protein	0.77	0.10
ADF	Crude protein	-0.94	0.02
Reducing sugar	Crude protein	0.66	0.18
NSP	Crude protein	-1.00	0.00
Ca	Crude protein	-0.75	0.08
P	Crude protein	0.88	0.02
Lactic acid	Crude protein	0.60	0.24
NDF	Crude protein	-0.83	0.06
Amylose	Crude protein	0.71	0.14
Starch	Crude protein	-0.66	0.18
Cellulose	Crude protein	-0.94	0.02
Crude protein	Ash	-0.94	0.02
Ash	Ash	1.00	0.00
Crude lipid	Ash	1.00	0.00
Acid-soluble protein	Ash	-0.83	0.06
ADF	Ash	1.00	0.00
Reducing sugar	Ash	-0.77	0.10
NSP	Ash	0.94	0.02
Ca	Ash	0.81	0.05
P	Ash	-0.79	0.06
Lactic acid	Ash	-0.71	0.14
NDF	Ash	0.77	0.10
Amylose	Ash	-0.77	0.10
Starch	Ash	0.77	0.10
Cellulose	Ash	1.00	0.00
Crude protein	Crude lipid	-0.94	0.02
Ash	Crude lipid	1.00	0.00
Crude lipid	Crude lipid	1.00	0.00
Acid-soluble protein	Crude lipid	-0.83	0.06
ADF	Crude lipid	1.00	0.00
Reducing sugar	Crude lipid	-0.77	0.10
NSP	Crude lipid	0.94	0.02
Ca	Crude lipid	0.81	0.05
P	Crude lipid	-0.79	0.06
Lactic acid	Crude lipid	-0.71	0.14

NDF	Crude lipid	0.77	0.10
Amylose	Crude lipid	-0.77	0.10
Starch	Crude lipid	0.77	0.10
Cellulose	Crude lipid	1.00	0.00
Crude protein	Acid-soluble protein	0.77	0.10
Ash	Acid-soluble protein	-0.83	0.06
Crude lipid	Acid-soluble protein	-0.83	0.06
Acid-soluble protein	Acid-soluble protein	1.00	0.00
ADF	Acid-soluble protein	-0.83	0.06
Reducing sugar	Acid-soluble protein	0.94	0.02
NSP	Acid-soluble protein	-0.77	0.10
Ca	Acid-soluble protein	-0.64	0.17
P	Acid-soluble protein	0.71	0.12
Lactic acid	Acid-soluble protein	0.89	0.03
NDF	Acid-soluble protein	-0.60	0.24
Amylose	Acid-soluble protein	0.77	0.10
Starch	Acid-soluble protein	-0.77	0.10
Cellulose	Acid-soluble protein	-0.83	0.06
Crude protein	ADF	-0.94	0.02
Ash	ADF	1.00	0.00
Crude lipid	ADF	1.00	0.00
Acid-soluble protein	ADF	-0.83	0.06
ADF	ADF	1.00	0.00
Reducing sugar	ADF	-0.77	0.10
NSP	ADF	0.94	0.02
Ca	ADF	0.81	0.05
P	ADF	-0.79	0.06
Lactic acid	ADF	-0.71	0.14
NDF	ADF	0.77	0.10
Amylose	ADF	-0.77	0.10
Starch	ADF	0.77	0.10
Cellulose	ADF	1.00	0.00
Crude protein	Reducing sugar	0.66	0.18
Ash	Reducing sugar	-0.77	0.10
Crude lipid	Reducing sugar	-0.77	0.10
Acid-soluble protein	Reducing sugar	0.94	0.02
ADF	Reducing sugar	-0.77	0.10
Reducing sugar	Reducing sugar	1.00	0.00
NSP	Reducing sugar	-0.66	0.18
Ca	Reducing sugar	-0.75	0.08
P	Reducing sugar	0.71	0.12

Lactic acid	Reducing sugar	0.94	0.02
NDF	Reducing sugar	-0.66	0.18
Amylose	Reducing sugar	0.89	0.03
Starch	Reducing sugar	-0.83	0.06
Cellulose	Reducing sugar	-0.77	0.10
Crude protein	NSP	-1.00	0.00
Ash	NSP	0.94	0.02
Crude lipid	NSP	0.94	0.02
Acid-soluble protein	NSP	-0.77	0.10
ADF	NSP	0.94	0.02
Reducing sugar	NSP	-0.66	0.18
NSP	NSP	1.00	0.00
Ca	NSP	0.75	0.08
P	NSP	-0.88	0.02
Lactic acid	NSP	-0.60	0.24
NDF	NSP	0.83	0.06
Amylose	NSP	-0.71	0.14
Starch	NSP	0.66	0.18
Cellulose	NSP	0.94	0.02
Crude protein	Ca	-0.75	0.08
Ash	Ca	0.81	0.05
Crude lipid	Ca	0.81	0.05
Acid-soluble protein	Ca	-0.64	0.17
ADF	Ca	0.81	0.05
Reducing sugar	Ca	-0.75	0.08
NSP	Ca	0.75	0.08
Ca	Ca	1.00	0.00
P	Ca	-0.85	0.03
Lactic acid	Ca	-0.75	0.08
NDF	Ca	0.93	0.01
Amylose	Ca	-0.90	0.01
Starch	Ca	0.84	0.04
Cellulose	Ca	0.81	0.05
Crude protein	P	0.88	0.02
Ash	P	-0.79	0.06
Crude lipid	P	-0.79	0.06
Acid-soluble protein	P	0.71	0.12
ADF	P	-0.79	0.06
Reducing sugar	P	0.71	0.12
NSP	P	-0.88	0.02
Ca	P	-0.85	0.03

P	P	1.00	0.00
Lactic acid	P	0.62	0.19
NDF	P	-0.97	0.00
Amylose	P	0.88	0.02
Starch	P	-0.62	0.19
Cellulose	P	-0.79	0.06
Crude protein	Lactic acid	0.60	0.24
Ash	Lactic acid	-0.71	0.14
Crude lipid	Lactic acid	-0.71	0.14
Acid-soluble protein	Lactic acid	0.89	0.03
ADF	Lactic acid	-0.71	0.14
Reducing sugar	Lactic acid	0.94	0.02
NSP	Lactic acid	-0.60	0.24
Ca	Lactic acid	-0.75	0.08
P	Lactic acid	0.62	0.19
Lactic acid	Lactic acid	1.00	0.00
NDF	Lactic acid	-0.60	0.24
Amylose	Lactic acid	0.77	0.10
Starch	Lactic acid	-0.94	0.02
Cellulose	Lactic acid	-0.71	0.14
Crude protein	NDF	-0.83	0.06
Ash	NDF	0.77	0.10
Crude lipid	NDF	0.77	0.10
Acid-soluble protein	NDF	-0.60	0.24
ADF	NDF	0.77	0.10
Reducing sugar	NDF	-0.66	0.18
NSP	NDF	0.83	0.06
Ca	NDF	0.93	0.01
P	NDF	-0.97	0.00
Lactic acid	NDF	-0.60	0.24
NDF	NDF	1.00	0.00
Amylose	NDF	-0.89	0.03
Starch	NDF	0.66	0.18
Cellulose	NDF	0.77	0.10
Crude protein	Amylose	0.71	0.14
Ash	Amylose	-0.77	0.10
Crude lipid	Amylose	-0.77	0.10
Acid-soluble protein	Amylose	0.77	0.10
ADF	Amylose	-0.77	0.10
Reducing sugar	Amylose	0.89	0.03
NSP	Amylose	-0.71	0.14

Ca	Amylose	-0.90	0.01
P	Amylose	0.88	0.02
Lactic acid	Amylose	0.77	0.10
NDF	Amylose	-0.89	0.03
Amylose	Amylose	1.00	0.00
Starch	Amylose	-0.71	0.14
Cellulose	Amylose	-0.77	0.10
Crude protein	Starch	-0.66	0.18
Ash	Starch	0.77	0.10
Crude lipid	Starch	0.77	0.10
Acid-soluble protein	Starch	-0.77	0.10
ADF	Starch	0.77	0.10
Reducing sugar	Starch	-0.83	0.06
NSP	Starch	0.66	0.18
Ca	Starch	0.84	0.04
P	Starch	-0.62	0.19
Lactic acid	Starch	-0.94	0.02
NDF	Starch	0.66	0.18
Amylose	Starch	-0.71	0.14
Starch	Starch	1.00	0.00
Cellulose	Starch	0.77	0.10
Crude protein	Cellulose	-0.94	0.02
Ash	Cellulose	1.00	0.00
Crude lipid	Cellulose	1.00	0.00
Acid-soluble protein	Cellulose	-0.83	0.06
ADF	Cellulose	1.00	0.00
Reducing sugar	Cellulose	-0.77	0.10
NSP	Cellulose	0.94	0.02
Ca	Cellulose	0.81	0.05
P	Cellulose	-0.79	0.06
Lactic acid	Cellulose	-0.71	0.14
NDF	Cellulose	0.77	0.10
Amylose	Cellulose	-0.77	0.10
Starch	Cellulose	0.77	0.10
Cellulose	Cellulose	1.00	0.00

Table S6 Spearman correlation coefficients (r) and P-values (P) between nutrient components and microbial taxa or metabolites

Row names	Column names	r	P
g__Lactobacillus	Crude protein	0.71	0.14
g__Romboutsia	Crude protein	0.53	0.28

g__HT002	Crude protein	0.93	0.01
g__Terrisporobacter	Crude protein	-0.77	0.10
g__Turicibacter	Crude protein	-0.71	0.14
g__Lactobacillus	Ash	-0.60	0.24
g__Romboutsia	Ash	-0.68	0.14
g__HT002	Ash	-0.93	0.01
g__Terrisporobacter	Ash	0.89	0.03
g__Turicibacter	Ash	0.77	0.10
g__Lactobacillus	Crude lipid	-0.60	0.24
g__Romboutsia	Crude lipid	-0.68	0.14
g__HT002	Crude lipid	-0.93	0.01
g__Terrisporobacter	Crude lipid	0.89	0.03
g__Turicibacter	Crude lipid	0.77	0.10
g__Lactobacillus	Acid-soluble protein	0.77	0.10
g__Romboutsia	Acid-soluble protein	0.79	0.06
g__HT002	Acid-soluble protein	0.93	0.01
g__Terrisporobacter	Acid-soluble protein	-0.89	0.03
g__Turicibacter	Acid-soluble protein	-0.60	0.24
g__Lactobacillus	ADF	-0.60	0.24
g__Romboutsia	ADF	-0.68	0.14
g__HT002	ADF	-0.93	0.01
g__Terrisporobacter	ADF	0.89	0.03
g__Turicibacter	ADF	0.77	0.10
g__Lactobacillus	Reducing sugar	0.71	0.14
g__Romboutsia	Reducing sugar	0.85	0.03
g__HT002	Reducing sugar	0.83	0.04
g__Terrisporobacter	Reducing sugar	-0.94	0.02
g__Turicibacter	Reducing sugar	-0.71	0.14
g__Lactobacillus	NSP	-0.71	0.14
g__Romboutsia	NSP	-0.53	0.28
g__HT002	NSP	-0.93	0.01
g__Terrisporobacter	NSP	0.77	0.10
g__Turicibacter	NSP	0.71	0.14
g__Lactobacillus	Ca	-0.64	0.17
g__Romboutsia	Ca	-0.72	0.11
g__HT002	Ca	-0.75	0.08
g__Terrisporobacter	Ca	0.84	0.04
g__Turicibacter	Ca	0.99	0.00
g__Lactobacillus	P	0.79	0.06
g__Romboutsia	P	0.48	0.33
g__HT002	P	0.81	0.05

g__Terrisporobacter	P	-0.79	0.06
g__Turicibacter	P	-0.79	0.06
g__Lactobacillus	Lactic acid	0.77	0.10
g__Romboutsia	Lactic acid	0.97	0.00
g__HT002	Lactic acid	0.83	0.04
g__Terrisporobacter	Lactic acid	-0.83	0.06
g__Turicibacter	Lactic acid	-0.77	0.10
g__Lactobacillus	NDF	-0.71	0.14
g__Romboutsia	NDF	-0.50	0.31
g__HT002	NDF	-0.74	0.09
g__Terrisporobacter	NDF	0.77	0.10
g__Turicibacter	NDF	0.89	0.03
g__Lactobacillus	Amylose	0.66	0.18
g__Romboutsia	Amylose	0.65	0.16
g__HT002	Amylose	0.74	0.09
g__Terrisporobacter	Amylose	-0.94	0.02
g__Turicibacter	Amylose	-0.83	0.06
g__Lactobacillus	Starch	-0.71	0.14
g__Romboutsia	Starch	-0.97	0.00
g__HT002	Starch	-0.83	0.04
g__Terrisporobacter	Starch	0.77	0.10
g__Turicibacter	Starch	0.89	0.03
g__Lactobacillus	Cellulose	-0.60	0.24
g__Romboutsia	Cellulose	-0.68	0.14
g__HT002	Cellulose	-0.93	0.01
g__Terrisporobacter	Cellulose	0.89	0.03
g__Turicibacter	Cellulose	0.77	0.10

Table S7 Spearman correlation coefficients (r) and P-values among metabolisms of the fermented substrate

Row names	Column names	r	P
Serine	Serine	1.00	0.00
Homoserine	Serine	0.94	0.02
N2,N2-Dimethylguanosine	Serine	0.94	0.02
Betonicine	Serine	0.77	0.10
Sarcosine	Serine	0.89	0.03
Alanine	Serine	0.89	0.03
beta-Alanine	Serine	0.89	0.03
Pisumionoside	Serine	0.77	0.10
Glutamate	Serine	0.94	0.02
vasicinone	Serine	0.77	0.10

metabolism1	Serine	-0.60	0.24
M261T51_1	Serine	-0.66	0.18
N-(4-hydroxybenzoyl)-glutamate	Serine	-0.66	0.18
agnuside	Serine	0.60	0.24
metabolism2	Serine	-0.66	0.18
Serine	Homoserine	0.94	0.02
Homoserine	Homoserine	1.00	0.00
N2,N2-Dimethylguanosine	Homoserine	0.89	0.03
Betonicine	Homoserine	0.66	0.18
Sarcosine	Homoserine	0.77	0.10
Alanine	Homoserine	0.77	0.10
beta-Alanine	Homoserine	0.77	0.10
Pisumionoside	Homoserine	0.83	0.06
Glutamate	Homoserine	0.89	0.03
vasicinone	Homoserine	0.66	0.18
metabolism1[1]	Homoserine	-0.54	0.30
M261T51_1	Homoserine	-0.60	0.24
N-(4-hydroxybenzoyl)-glutamate	Homoserine	-0.60	0.24
agnuside	Homoserine	0.66	0.18
metabolism2[2]	Homoserine	-0.60	0.24
Serine	N2,N2-Dimethylguanosine	0.94	0.02
Homoserine	N2,N2-Dimethylguanosine	0.89	0.03
N2,N2-Dimethylguanosine	N2,N2-Dimethylguanosine	1.00	0.00
Betonicine	N2,N2-Dimethylguanosine	0.71	0.14
Sarcosine	N2,N2-Dimethylguanosine	0.94	0.02
Alanine	N2,N2-Dimethylguanosine	0.94	0.02
beta-Alanine	N2,N2-Dimethylguanosine	0.94	0.02
Pisumionoside	N2,N2-Dimethylguanosine	0.71	0.14
Glutamate	N2,N2-Dimethylguanosine	1.00	0.00
vasicinone	N2,N2-Dimethylguanosine	0.71	0.14
metabolism1	N2,N2-Dimethylguanosine	-0.66	0.18
M261T51_1	N2,N2-Dimethylguanosine	-0.77	0.10
N-(4-hydroxybenzoyl)-glutamate	N2,N2-Dimethylguanosine	-0.60	0.24
agnuside	N2,N2-Dimethylguanosine	0.54	0.30
metabolism2	N2,N2-Dimethylguanosine	-0.77	0.10
Serine	Betonicine	0.77	0.10
Homoserine	Betonicine	0.66	0.18
N2,N2-Dimethylguanosine	Betonicine	0.71	0.14
Betonicine	Betonicine	1.00	0.00
Sarcosine	Betonicine	0.77	0.10
Alanine	Betonicine	0.77	0.10

beta-Alanine	Betonicine	0.77	0.10
Pisumionoside	Betonicine	0.66	0.18
Glutamate	Betonicine	0.71	0.14
vasicinone	Betonicine	1.00	0.00
metabolism1	Betonicine	-0.89	0.03
M261T51_1	Betonicine	-0.77	0.10
N-(4-hydroxybenzoyl)-glutamate	Betonicine	-0.94	0.02
agnuside	Betonicine	0.83	0.06
metabolism2	Betonicine	-0.77	0.10
Serine	Sarcosine	0.89	0.03
Homoserine	Sarcosine	0.77	0.10
N2,N2-Dimethylguanosine	Sarcosine	0.94	0.02
Betonicine	Sarcosine	0.77	0.10
Sarcosine	Sarcosine	1.00	0.00
Alanine	Sarcosine	1.00	0.00
beta-Alanine	Sarcosine	1.00	0.00
Pisumionoside	Sarcosine	0.77	0.10
Glutamate	Sarcosine	0.94	0.02
vasicinone	Sarcosine	0.77	0.10
metabolism1	Sarcosine	-0.77	0.10
M261T51_1	Sarcosine	-0.89	0.03
N-(4-hydroxybenzoyl)-glutamate	Sarcosine	-0.71	0.14
agnuside	Sarcosine	0.60	0.24
metabolism2	Sarcosine	-0.89	0.03
Serine	Alanine	0.89	0.03
Homoserine	Alanine	0.77	0.10
N2,N2-Dimethylguanosine	Alanine	0.94	0.02
Betonicine	Alanine	0.77	0.10
Sarcosine	Alanine	1.00	0.00
Alanine	Alanine	1.00	0.00
beta-Alanine	Alanine	1.00	0.00
Pisumionoside	Alanine	0.77	0.10
Glutamate	Alanine	0.94	0.02
vasicinone	Alanine	0.77	0.10
metabolism1	Alanine	-0.77	0.10
M261T51_1	Alanine	-0.89	0.03
N-(4-hydroxybenzoyl)-glutamate	Alanine	-0.71	0.14
agnuside	Alanine	0.60	0.24
metabolism2	Alanine	-0.89	0.03
Serine	beta-Alanine	0.89	0.03
Homoserine	beta-Alanine	0.77	0.10

N2,N2-Dimethylguanosine	beta-Alanine	0.94	0.02
Betonicine	beta-Alanine	0.77	0.10
Sarcosine	beta-Alanine	1.00	0.00
Alanine	beta-Alanine	1.00	0.00
beta-Alanine	beta-Alanine	1.00	0.00
Pisumionoside	beta-Alanine	0.77	0.10
Glutamate	beta-Alanine	0.94	0.02
vasicinone	beta-Alanine	0.77	0.10
metabolism1	beta-Alanine	-0.77	0.10
M261T51_1	beta-Alanine	-0.89	0.03
N-(4-hydroxybenzoyl)-glutamate	beta-Alanine	-0.71	0.14
agnuside	beta-Alanine	0.60	0.24
metabolism2	beta-Alanine	-0.89	0.03
Serine	Pisumionoside	0.77	0.10
Homoserine	Pisumionoside	0.83	0.06
N2,N2-Dimethylguanosine	Pisumionoside	0.71	0.14
Betonicine	Pisumionoside	0.66	0.18
Sarcosine	Pisumionoside	0.77	0.10
Alanine	Pisumionoside	0.77	0.10
beta-Alanine	Pisumionoside	0.77	0.10
Pisumionoside	Pisumionoside	1.00	0.00
Glutamate	Pisumionoside	0.71	0.14
vasicinone	Pisumionoside	0.66	0.18
metabolism1	Pisumionoside	-0.71	0.14
M261T51_1	Pisumionoside	-0.77	0.10
N-(4-hydroxybenzoyl)-glutamate	Pisumionoside	-0.77	0.10
agnuside	Pisumionoside	0.83	0.06
metabolism2	Pisumionoside	-0.77	0.10
Serine	Glutamate	0.94	0.02
Homoserine	Glutamate	0.89	0.03
N2,N2-Dimethylguanosine	Glutamate	1.00	0.00
Betonicine	Glutamate	0.71	0.14
Sarcosine	Glutamate	0.94	0.02
Alanine	Glutamate	0.94	0.02
beta-Alanine	Glutamate	0.94	0.02
Pisumionoside	Glutamate	0.71	0.14
Glutamate	Glutamate	1.00	0.00
vasicinone	Glutamate	0.71	0.14
metabolism1	Glutamate	-0.66	0.18
M261T51_1	Glutamate	-0.77	0.10
N-(4-hydroxybenzoyl)-glutamate	Glutamate	-0.60	0.24

agnuside	Glutamate	0.54	0.30
metabolism2	Glutamate	-0.77	0.10
Serine	vasicinone	0.77	0.10
Homoserine	vasicinone	0.66	0.18
N2,N2-Dimethylguanosine	vasicinone	0.71	0.14
Betonicine	vasicinone	1.00	0.00
Sarcosine	vasicinone	0.77	0.10
Alanine	vasicinone	0.77	0.10
beta-Alanine	vasicinone	0.77	0.10
Pisumionoside	vasicinone	0.66	0.18
Glutamate	vasicinone	0.71	0.14
vasicinone	vasicinone	1.00	0.00
metabolism1	vasicinone	-0.89	0.03
M261T51_1	vasicinone	-0.77	0.10
N-(4-hydroxybenzoyl)-glutamate	vasicinone	-0.94	0.02
agnuside	vasicinone	0.83	0.06
metabolism2	vasicinone	-0.77	0.10
Serine	metabolism1	-0.60	0.24
Homoserine	metabolism1	-0.54	0.30
N2,N2-Dimethylguanosine	metabolism1	-0.66	0.18
Betonicine	metabolism1	-0.89	0.03
Sarcosine	metabolism1	-0.77	0.10
Alanine	metabolism1	-0.77	0.10
beta-Alanine	metabolism1	-0.77	0.10
Pisumionoside	metabolism1	-0.71	0.14
Glutamate	metabolism1	-0.66	0.18
vasicinone	metabolism1	-0.89	0.03
metabolism1	metabolism1	1.00	0.00
M261T51_1	metabolism1	0.94	0.02
N-(4-hydroxybenzoyl)-glutamate	metabolism1	0.94	0.02
agnuside	metabolism1	-0.89	0.03
metabolism2	metabolism1	0.94	0.02
Serine	M261T51_1	-0.66	0.18
Homoserine	M261T51_1	-0.60	0.24
N2,N2-Dimethylguanosine	M261T51_1	-0.77	0.10
Betonicine	M261T51_1	-0.77	0.10
Sarcosine	M261T51_1	-0.89	0.03
Alanine	M261T51_1	-0.89	0.03
beta-Alanine	M261T51_1	-0.89	0.03
Pisumionoside	M261T51_1	-0.77	0.10
Glutamate	M261T51_1	-0.77	0.10

vasicinone	M261T51_1	-0.77	0.10
metabolism1	M261T51_1	0.94	0.02
M261T51_1	M261T51_1	1.00	0.00
N-(4-hydroxybenzoyl)-glutamate	M261T51_1	0.83	0.06
agnuside	M261T51_1	-0.77	0.10
metabolism2	M261T51_1	1.00	0.00
Serine	N-(4-hydroxybenzoyl)-glutamate	-0.66	0.18
Homoserine	N-(4-hydroxybenzoyl)-glutamate	-0.60	0.24
N2,N2-Dimethylguanosine	N-(4-hydroxybenzoyl)-glutamate	-0.60	0.24
Betonicine	N-(4-hydroxybenzoyl)-glutamate	-0.94	0.02
Sarcosine	N-(4-hydroxybenzoyl)-glutamate	-0.71	0.14
Alanine	N-(4-hydroxybenzoyl)-glutamate	-0.71	0.14
beta-Alanine	N-(4-hydroxybenzoyl)-glutamate	-0.71	0.14
Pisumionoside	N-(4-hydroxybenzoyl)-glutamate	-0.77	0.10
Glutamate	N-(4-hydroxybenzoyl)-glutamate	-0.60	0.24
vasicinone	N-(4-hydroxybenzoyl)-glutamate	-0.94	0.02
metabolism1	N-(4-hydroxybenzoyl)-glutamate	0.94	0.02
M261T51_1	N-(4-hydroxybenzoyl)-glutamate	0.83	0.06
N-(4-hydroxybenzoyl)-glutamate	N-(4-hydroxybenzoyl)-glutamate	1.00	0.00
agnuside	N-(4-hydroxybenzoyl)-glutamate	-0.94	0.02
metabolism2	N-(4-hydroxybenzoyl)-glutamate	0.83	0.06
Serine	agnuside	0.60	0.24
Homoserine	agnuside	0.66	0.18
N2,N2-Dimethylguanosine	agnuside	0.54	0.30
Betonicine	agnuside	0.83	0.06
Sarcosine	agnuside	0.60	0.24
Alanine	agnuside	0.60	0.24
beta-Alanine	agnuside	0.60	0.24
Pisumionoside	agnuside	0.83	0.06
Glutamate	agnuside	0.54	0.30
vasicinone	agnuside	0.83	0.06
metabolism1	agnuside	-0.89	0.03
M261T51_1	agnuside	-0.77	0.10
N-(4-hydroxybenzoyl)-glutamate	agnuside	-0.94	0.02
agnuside	agnuside	1.00	0.00
metabolism2	agnuside	-0.77	0.10
Serine	metabolism2	-0.66	0.18
Homoserine	metabolism2	-0.60	0.24
N2,N2-Dimethylguanosine	metabolism2	-0.77	0.10
Betonicine	metabolism2	-0.77	0.10
Sarcosine	metabolism2	-0.89	0.03

Alanine	metabolism2	-0.89	0.03
beta-Alanine	metabolism2	-0.89	0.03
Pisumionoside	metabolism2	-0.77	0.10
Glutamate	metabolism2	-0.77	0.10
vasicinone	metabolism2	-0.77	0.10
metabolism1	metabolism2	0.94	0.02
M261T51_1	metabolism2	1.00	0.00
N-(4-hydroxybenzoyl)-glutamate	metabolism2	0.83	0.06
agnuside	metabolism2	-0.77	0.10
metabolism2	metabolism2	1.00	0.00

metabolism1[1]: 2-[4-[3-[3,4-dihydroxy-4-(hydroxymethyl) oxolan-2-yl] oxy-4,5-dihydroxy-6-(hydroxymethyl) oxan-2-yl] oxyphenyl]-7-hydroxy-2,3-dihydrochromen-4-one

metabolism2[2]: [(2R,3S,4R,5R,6R)-6-[2-(3,4-dihydroxyphenyl) ethoxy]-4,5-dihydroxy-2-[[(2S,3R,4S,5R)-3,4,5-trihydroxyoxan-2-yl] oxymethyl] oxan-3-yl] (E)-3-(3,4-dihydroxyphenyl) prop-2-enoate

Table S8 Spearman correlation coefficients (r) and P-values (P) between microbiota and metabolites

Row names	Column names	r	P
g__Lactobacillus	Serine	0.94	0.02
g__Romboutsia	Serine	0.74	0.10
g__HT002	Serine	0.74	0.09
g__Terrisporobacter	Serine	-0.66	0.18
g__Turicibacter	Serine	-0.77	0.10
g__Lactobacillus	Homoserine	0.83	0.06
g__Romboutsia	Homoserine	0.88	0.02
g__HT002	Homoserine	0.74	0.09
g__Terrisporobacter	Homoserine	-0.77	0.10
g__Turicibacter	Homoserine	-0.83	0.06
g__Lactobacillus	N2,N2-Dimethylguanosine	0.89	0.03
g__Romboutsia	N2,N2-Dimethylguanosine	0.74	0.10
g__HT002	N2,N2-Dimethylguanosine	0.74	0.09
g__Terrisporobacter	N2,N2-Dimethylguanosine	-0.60	0.24
g__Turicibacter	N2,N2-Dimethylguanosine	-0.89	0.03
g__Lactobacillus	Betonicine	0.83	0.06
g__Romboutsia	Betonicine	0.44	0.38
g__HT002	Betonicine	0.83	0.04
g__Terrisporobacter	Betonicine	-0.77	0.10
g__Turicibacter	Betonicine	-0.66	0.18
g__Lactobacillus	Sarcosine	0.94	0.02
g__Romboutsia	Sarcosine	0.68	0.14
g__HT002	Sarcosine	0.83	0.04

g__Terrisporobacter	Sarcosine	-0.54	0.30
g__Turicibacter	Sarcosine	-0.77	0.10
g__Lactobacillus	Alanine	0.94	0.02
g__Romboutsia	Alanine	0.68	0.14
g__HT002	Alanine	0.83	0.04
g__Terrisporobacter	Alanine	-0.54	0.30
g__Turicibacter	Alanine	-0.77	0.10
g__Lactobacillus	beta-Alanine	0.94	0.02
g__Romboutsia	beta-Alanine	0.68	0.14
g__HT002	beta-Alanine	0.83	0.04
g__Terrisporobacter	beta-Alanine	-0.54	0.30
g__Turicibacter	beta-Alanine	-0.77	0.10
g__Lactobacillus	Pisumionoside	0.83	0.06
g__Romboutsia	Pisumionoside	0.91	0.01
g__HT002	Pisumionoside	0.93	0.01
g__Terrisporobacter	Pisumionoside	-0.77	0.10
g__Turicibacter	Pisumionoside	-0.66	0.18
g__Lactobacillus	Glutamate	0.89	0.03
g__Romboutsia	Glutamate	0.74	0.10
g__HT002	Glutamate	0.74	0.09
g__Terrisporobacter	Glutamate	-0.60	0.24
g__Turicibacter	Glutamate	-0.89	0.03
g__Lactobacillus	vasicinone	0.83	0.06
g__Romboutsia	vasicinone	0.44	0.38
g__HT002	vasicinone	0.83	0.04
g__Terrisporobacter	vasicinone	-0.77	0.10
g__Turicibacter	vasicinone	-0.66	0.18
g__Lactobacillus	metabolism1[1]	-0.71	0.14
g__Romboutsia	metabolism1	-0.53	0.28
g__HT002	metabolism1	-0.93	0.01
g__Terrisporobacter	metabolism1	0.77	0.10
g__Turicibacter	metabolism1	0.71	0.14
g__Lactobacillus	M261T51_1	-0.77	0.10
g__Romboutsia	M261T51_1	-0.65	0.16
g__HT002	M261T51_1	-0.93	0.01
g__Terrisporobacter	M261T51_1	0.66	0.18
g__Turicibacter	M261T51_1	0.77	0.10
g__Lactobacillus	N-(4-hydroxybenzoyl)-glutamate	-0.77	0.10
g__Romboutsia	N-(4-hydroxybenzoyl)-glutamate	-0.53	0.28
g__HT002	N-(4-hydroxybenzoyl)-glutamate	-0.93	0.01
g__Terrisporobacter	N-(4-hydroxybenzoyl)-glutamate	0.83	0.06

g__Turicibacter	N-(4-hydroxybenzoyl)-glutamate	0.60	0.24
g__Lactobacillus	agnuside	0.66	0.18
g__Romboutsia	agnuside	0.68	0.14
g__HT002	agnuside	0.93	0.01
g__Terrisporobacter	agnuside	-0.94	0.02
g__Turicibacter	agnuside	-0.66	0.18
g__Lactobacillus	metabolism2[2]	-0.77	0.10
g__Romboutsia	metabolism2	-0.65	0.16
g__HT002	metabolism2	-0.93	0.01
g__Terrisporobacter	metabolism2	0.66	0.18
g__Turicibacter	metabolism2	0.77	0.10

metabolism1[1]: 2-[4-[3-[3,4-dihydroxy-4-(hydroxymethyl) oxolan-2-yl] oxy-4,5-dihydroxy-6-(hydroxymethyl) oxan-2-yl] oxyphenyl]-7-hydroxy-2,3-dihydrochromen-4-one

metabolism2[2]: [(2R,3S,4R,5R,6R)-6-[2-(3,4-dihydroxyphenyl) ethoxy]-4,5-dihydroxy-2-[[(2S,3R,4S,5R)-3,4,5-trihydroxyoxan-2-yl] oxymethyl] oxan-3-yl] (E)-3-(3,4-dihydroxyphenyl) prop-2-enoate
